# In-Hospital Outcomes of Coronary Artery Stenting in Patients With ST-Elevation Myocardial Infarction (STEMI) and Metabolic Syndrome: Insights From the National Inpatient Sample

**DOI:** 10.7759/cureus.24664

**Published:** 2022-05-02

**Authors:** Owen Igbinosa, Ahmed Brgdar, Joseph Asemota, Mohamed E Taha, Jin Yi, Anthony Lyonga Ngonge, Swati Vanaparthy, Raccquel Hammonds, Joseph Talbet, Diannemarie Omire-Mayor, Julius Ngwa, Muhammad Rizwan, Mehrotra Prafulla, Isaac Opoku

**Affiliations:** 1 Internal Medicine, Howard University Hospital, Washington, DC, USA; 2 Cardiovascular Medicine, Howard University, Washington, DC, USA; 3 Plastic and Reconstructive Surgery, Howard University Hospital, Washington, DC, USA; 4 Internal Medicine, Howard University College of Medicine, Washington, DC, USA; 5 Cardiovascular Disease, Howard University Hospital, Washington, DC, USA

**Keywords:** percutaneous coronary intervention, stemi, obesity paradox, metabolic syndrome, mortality

## Abstract

Background

Metabolic syndrome (MetS) has been recognized as a global health problem. Concurrent MetS diagnosis in patients with ST-elevation myocardial infarction (STEMI) is becoming increasingly common. Given the paucity of studies on the impact of MetS on treatment outcomes in STEMI patients, the purpose of this study was to evaluate in-hospital mortality in STEMI patients with a concurrent MetS diagnosis undergoing a stenting procedure to treat their underlying coronary artery disease.

Method

Patients with or without MetS who underwent coronary stenting following STEMI between 2005 and 2014 were identified from the National Inpatient Sample database. Patients' demographics, comorbidities, and outcomes were compared using a t-test and Pearson's Chi-square test. In addition, 1:1 propensity score matching was performed for age, gender, and race.

Results

Out of 1,938,097 STEMI patients, 5,817 patients with MetS underwent coronary stenting following STEMI and were matched with 5,817 patients with no Mets. MetS group had significantly higher rates of diabetes, hypertension, hyperlipidemia, chronic kidney disease, and obstructive sleep apnea than the no MetS group but lower rates of heart failure and chronic obstructive pulmonary disease. In-hospital mortality following STEMI was significantly lower in patients with MetS (2.5% vs. 7.1%, p<0.001) and remained significant after adjusting for potential confounders (odds ratio (OR) 0.34, 95% confidence interval (95% CI) 0.28-0.42, p<0.0001).

Conclusion

Concurrent diagnosis of MetS among patients undergoing coronary stenting is associated with a decreased in-hospital mortality risk. The impact of specific MetS components on the observed reduction in mortality remains unclear and warrants evaluation in future studies.

## Introduction

Metabolic syndrome (MetS) is a serious health problem with a steadily increasing prevalence. Among the US adult population, the prevalence of the metabolic syndrome is currently estimated at approximately 37% [1], and it is expected to rise further with the global obesity epidemic. MetS is characterized by a constellation of obesity-related conditions, including elevated blood pressure, hyperglycemia, and imbalances in the levels of lipoproteins and cholesterol (C), with independent risk effects that together become synergistic and impose major risk on cardiovascular health, ultimately resulting in a higher risk of all-cause and cardiac mortality [2-5].

The prevalence of MetS may be disproportionately higher (40-70%) among patients with ST-elevation myocardial infarction (STEMI) [6-8]. The existing literature suggests that metabolic syndrome invariably causes damage to the microcirculation of the heart following direct percutaneous coronary intervention (PCI), ultimately leading to a poor prognosis [9-11]. Consequently, STEMI patients with MetS have an exacerbated risk of major adverse cardiac events (MACEs), especially new revascularization [11,12]. Further, MetS has been associated with an increased occurrence of the no-flow phenomenon among STEMI patients treated with PCI [13]. However, there is increasing recognition of a phenomenon referred to as the “obesity paradox” wherein better cardiovascular disease (CVD) outcomes are demonstrated in overweight and at least mildly obese patients [14]. Congruent with these observations, MetS does not seem to increase the risk of all-cause mortality, including in-hospital mortality, among patients with STEMI compared to those without MetS [11,12,15].

Although stenting procedures are increasingly being used to treat MetS patients with coronary obstructions [16,17], especially with drug-eluting stents (DES) due to their superior clinical outcomes over conventional bare-metal stents (BMS) [18], the efficacy and short-term mortality outcomes of stent implantation among myocardial infarction (MI) patients with or without MetS are not known. Moreover, most prior studies have not differentiated between different MI types-STEMI vs. non-STEMI (NSTEMI)-while investigating the association between MetS and mortality outcomes. Therefore, the primary purpose of this study was to assess the in-hospital mortality among STEMI patients with MetS who underwent a stenting procedure using data from the extensive National Inpatient Sample (NIS) database.

## Materials and methods

Data source

The NIS is an Agency for Healthcare Research and Quality (AHRQ) sponsored public inpatient healthcare database developed and maintained by the Healthcare Cost and Utilization Project (HCUP) that can be utilized to provide national estimates of health care utilization, cost, quality, and outcomes [19]. The NIS database records over seven million individual hospitalizations annually and includes the principal diagnosis (primary discharge diagnosis), medical procedures performed during hospitalization, total hospital costs, length of stay, and up to 29 secondary diagnoses [19]. In the United States, results from the NIS have been shown to correlate well with other hospitalization and discharge databases.

Study population

Patients ≥18 years of age who underwent coronary artery stenting following STEMI in the NIS database from 2005 to 2014 were identified using the International Classification of Diseases, ninth diagnosis code (ICD-9). The ICD-9 codes 410.0x, 410.1x, 410.2x, 410.3x, 410.4x, 410.5x, 410.6x, and 410.8x, 410.9 corresponding to STEMI, 36.07, and 36.06 corresponding to DES and BMS, respectively, were used to identify the population sample for this study. Data were obtained on patient characteristics and hospital-level characteristics. Baseline patient characteristics included age, gender, race/ethnicity, income, insurance type, length of hospitalization, and comorbidities. Hospital characteristics included location (urban and rural) and teaching status. Subsequently, the sample was dichotomized into two groups: STEMI patients with MetS (ICD-9 = 277.7 corresponding to metabolic syndrome) and STEMI patients without MetS. Records with incomplete data for mortality, concomitant coronary artery bypass graft, vascular surgery, or valvular heart surgery were excluded.

Outcomes

The primary outcome was in-hospital mortality following coronary stenting in STEMI patients with metabolic syndrome. Secondary outcomes included length and cost of hospitalization. In addition, demographic and comorbid factors were identified as covariates.

Analysis

Categorical data are presented as a frequency in percentage, and continuous data are presented as mean (SD). Differences between categorical variables were evaluated using the χ^2^ test, and differences between continuous variables were tested using the Student's t-test. The Wilcoxon rank-sum test was used to compare continuous variables for skewed and normal distribution data where appropriate. To remove selection bias from our study, we performed a propensity score-matched analysis. First, a logistic regression model was performed to calculate each patient's propensity score. Then, we matched all patients using a 1:1 scheme without replacement using the nearest number matching method. In our propensity model, we included age, sex, and race. Multivariate logistic regression was used to calculate adjusted odds ratios (aOR) and 95% confidence intervals (95% CI) for the association between MetS status and primary and secondary study outcomes. Data analysis was performed with Stata software (StataCorp LP, College Station, TX). All statistical tests were two-sided, and tests with p<0.05 were considered significant.

## Results

From 2005 to 2014, 1,938,097 in-hospital records of patients with STEMI diagnoses were retrieved from the NIS database. 5,817 patients with MetS who underwent coronary stenting following STEMI were compared to 5,817 age-, sex-, and race-matched patients with no MetS who also underwent coronary stenting following STEMI (Table 1). Most patients were male (65.6%) and Caucasian (77.2%), with no difference between groups (Table 1). The coronary stenting procedures were mainly performed at urban teaching hospitals (46.5%), and Medicare was the primary payer for most patients (41.95%) (Table 1). Baseline differences in comorbidities existed between the groups: the MetS group had significantly higher rates of diabetes mellitus (58.2% vs. 34.2%), hypertension (80.8% vs. 66.3%), hyperlipidemia (77.1% vs. 53.4%), chronic kidney disease (CKD; 17.3% vs. 15.5%), and obstructive sleep apnea (12.9% vs. 4.6%) compared to the no MetS group, but lower rates of heart failure (26.7% vs. 28.5%) and chronic obstructive pulmonary disease (COPD; 14.9% vs. 17.4%) (all p-values <0.05; Table 1). Also, the MetS group had fewer smokers (25.7% vs. 28.5%) and alcohol users (1.6% vs. 2.6%) (Table 1).

**Table 1:** Characteristics of STEMI patients with or without MetS who underwent coronary stenting between 2005 and 2014. STEMI: ST-elevation myocardial infarction; MetS: metabolic syndrome.

Variables	MetS (n = 5,817)	No MetS (n = 5,817)	p-value
Mean age (SD)	60.45 (12.75)	60.45 (12.74)	0.995
Gender			
Male	3,816 (65.6%)	3,815 (65.6%)	1.000
Female	2,001 (34.4%)	2,002 (34.4%)	
Race			
White	4,493 (77.2%)	4,493 (77.2%)	1.000
Black	471 (8.1%)	470 (8.1%)	
Hispanics	486 (8.4%)	486 (8.4%)	
Asian or Pacific Islander	136 (2.3%)	136 (2.3%)	
Native Americans	45 (0.8%)	46 (0.8%)	
Others	186 (3.2%)	186 (3.2%)	
Median income			
$1–$38,999	1,457 (25.7%)	1,661 (29.4%)	<0.0001
$39,000–$47,999	1,553 (27.3%)	1,539 (27.3%)	
$48,000–$62,999	1,459 (25.7%)	1,360 (24.1%)	
$63,000 or more	1,211 (21.3%)	1.083 (19.2%)	
Location and teaching status			
Rural	412 (7.1%)	534 (9.2%)	<0.0001
Urban non-teaching	2,836 (49.0%)	2,416 (41.7%)	
Urban teaching	2,537 (43.9%)	2,846 (49.1%)	
Insurance			
Medicare	2,392 (41.2%)	2,481 (42.7%)	<0.0001
Medicaid	432 (7.4%)	543 (9.4%)	
Private insurance	2,313 (39.8%)	2,036 (35.1%)	
Self-pay	412 (7.1%)	505 (8.7%)	
No charge	54 (0.9%)	50 (0.9%)	
Other	206 (3.5%)	192 (3.3%)	
Hospital regions			
Northeast	803 (13.8%)	1,289 (22.2%)	
Midwest	1,413 (57.3%)	1,053 (42.7%)	
South	2,335 (40.1%)	2,416 (41.5%)	
West	1,266 (21.8%)	1,059 (18.2%)	
Comorbidities			
Diabetes mellitus	3,386 (58.2%)	1,989 (34.2%)	<0.0001
Hypertension	4,698 (80.8%)	3,857 (66.3%)	<0.0001
Hyperlipidemia	4,486 (77.1%)	3,104 (53.4%)	<0.0001
Heart failure	1,554 (26.7%)	1,655 (28.5%)	0.038
Chronic kidney disease	1,004 (17.3%)	900 (15.5%)	0.010
Chronic obstructive pulmonary disease	869 (14.9%)	1,014 (17.4%)	0.0003
Atrial fibrillation	776 (13.3%)	728 (12.5%)	0.194
Obstructive sleep apnea	748 (12.9%)	267 (4.6%)	<0.0001
Anemia	1,034 (17.8%)	926 (15.9%)	0.008
Smoking	1,493 (25.7%)	1,658 (28.5%)	0.0006
Alcohol	92 (1.6%)	153 (2.6%)	<0.0001

In our univariate analysis, in-hospital mortality following STEMI was significantly lower in patients with the MetS group compared to the non-MetS group (2.5% vs. 7.1%, p<0.001) (Table 2). The median length of hospitalization was similar in both groups (three days, p = 0.590), but the median total cost of hospitalization was 7.35% lower in the MetS group (p = <0.0001) (Table 2).

**Table 2:** Hospital death, length of hospitalization, and total cost among STEMI patients with or without MetS who underwent coronary stenting between 2005 and 2014. STEMI: ST-elevation myocardial infarction; MetS: metabolic syndrome.

		MetS (N = 5,817)	No MetS (N = 5,817)	p-value
Hospital death	No	5,674 (97.5%)	5,401 (92.9%)	<0.0001
	Yes	143 (2.5%)	414 (7.1%)	
Length of hospitalization (range)		3 (2, 6)	3 (2, 7)	0.590
Total hospital cost (range)		54,922 (31,137, 92,157)	59,114 (27,766, 86,880)	<0.0001

After adjusting for potential confounders, including baseline characteristics, comorbidities, and hospital characteristics, the MetS group had significantly lower odds of in-hospital mortality (aOR 0.34, 95% CI 0.27-0.40, p<0.0001) and a lower mean length of hospitalization (beta −0.57±0.13, t = −4.44, p<0.0001) (Table 3). After adjusting for confounding variables, there was, however, no significant difference in the total cost of hospitalization (Table 3).

**Table 3:** Unadjusted and adjusted association of hospital death, length of hospitalization, and total cost among STEMI patients with MetS who underwent coronary stenting between 2005 and 2014. STEMI: ST-elevation myocardial infarction; MetS: metabolic syndrome. ^a^SE: standard error.

	MetS vs. No MetS							
	Adjusted analysis			Unadjusted analysis				
Logistic model	OR (95% CI)		p	OR (95% CI)				p
Hospital death	0.33 (0.27–0.40)		<0.0001	0.40 (0.32–0.50)				<0.0001
Generalized linear model	Beta	SE^a^	p	Beta	SE^a^	p		
Length of hospitalization	−0.70	0.08	<0.0001	−0.57	0.13		<0.0001	
Total hospital cost	902.92	1,518.26	0.552	−1,856.08	1,700.64		0.275	

Among patients with MetS, concurrent diagnosis of diabetes mellitus or obstructive sleep apnea was not associated with hospital death (p = 0.865) but was associated with a shorter length of hospitalization (p<0.0001), albeit with a higher total hospital cost (p<0.0001) (Table 4). On the other hand, the diagnosis of CKD or COPD was associated with an increased risk of hospital death, a longer length of hospitalization, and greater total cost (all p<0.05) (Table 4). Hypertension was associated with a lower risk of hospital death (p = 0.004) and a shorter length of hospitalization (p = 0.005) (Table 4).

**Table 4:** In-hospital outcomes and clinical risk factors among STEMI patients with or without MetS who underwent coronary stenting between 2005 and 2014. COPD: chronic obstructive pulmonary disease; STEMI: ST-elevation myocardial infarction; MetS: metabolic syndrome. ^a^SE: standard error.

	Clinical risk factors	Hospital death		Length of hospitalization			Total hospital cost		
		OR (95% CI)	p	Beta	SE^a^	p	Beta	SE^a^	p
MetS	Diabetes mellitus	1.03 (0.71–1.49)	0.865	0.81	0.13	<0.0001	9,417.13	2,095.99	<0.0001
	Hypertension	0.54 (0.36–0.82)	0.004	−0.45	0.16	0.005	−512.13	2,589.54	0.843
	Chronic kidney disease	2.02 (1.37–2.97)	0.0003	2.39	0.18	<0.0001	15,069.25	2,833.90	<0.0001
	COPD	1.66 (1.13–2.44)	0.010	1.16	0.18	<0.0001	9,854.14	2,880.30	0.001
	Obstructive sleep apnea	0.94 (0.55–1.59)	0.811	0.65	0.19	0.001	11,874.52	3,045.26	<0.0001
	Alcohol	0.77 (0.10–5.72)	0.796	0.44	0.52	0.396	9,866.87	8,300.97	0.235
No MetS	Diabetes mellitus	0.70 (0.55–0.899)	0.005	0.04	0.22	0.854	−2.83	2,546.00	0.999
	Hypertension	0.49 (0.39–0.619)	<0.0001	−1.54	0.21	<0.0001	−12,264.02	2,520.54	<0.0001
	Chronic kidney disease	2.07 (1.58–2.698)	<0.0001	2.05	0.28	<0.0001	15,890.67	3,358.62	<0.0001
	COPD	1.61 (1.26–2.053)	<0.0001	1.47	0.26	< 0.0001	9,788.45	3,058.44	0.001
	Obstructive sleep apnea	0.79 (0.44–1.445)	0.449	0.57	0.47	0.228	2,636.65	5,598.95	0.638
	Alcohol	0.80 (0.34–1.850)	0.599	−0.37	0.62	0.550	−1,451.58	7,359.16	0.844
	Obesity	0.72 (0.49–1.066)	0.102	−0.57	0.29	0.049	342.81	3,418.59	0.920

In contrast, concurrent diagnosis of diabetes mellitus in the no MetS group was associated with a lower risk of hospital death (p = 0.005), while the presence of CKD or COPD was associated with an increased risk of hospital death (p<0.0001), a longer length of hospitalization (p<0.0001), and greater total cost (p<0.0001 in CKD, p = 0.001 in COPD) (Table 4). However, like in the MetS group, hypertension was associated with a lower risk of hospital death (p<0.0001) and shorter length of hospitalization (p<0.0001) in the no MetS group, in addition to a lower total cost (p<0.0001) (Table 4).

## Discussion

The impact of MetS on mortality outcomes has been inconsistently reported. While the Gruppo Italiano per lo Studio della Sopravvivenza nell'Infarto Miocardio (GISSI)-Prevenzione trial reported an increased risk of all-cause death/mortality in MI patients with MetS [20], a four-year longitudinal study by Lovic et al. in Serbia found no association between MetS and increased mortality among STEMI patients [12]. In European cohorts of MI patients, MetS was identified as an independent predictor of severe heart failure [15], MACE [11], and coronary artery disease (CAD) [11] but was not associated with in-hospital mortality [11,15]. In contrast, an analysis of the Korea Acute Myocardial Infarction Registry showed that the diagnosis of MetS among patients with acute STEMI was associated with more significant in-hospital mortality, even though the length of hospitalization and MACE occurrence within 12 months of follow-up was similar to patients without MetS [21]. However, none of these studies specifically recruited patients undergoing coronary stent implantation, as was done in our study.

Despite the well-established fact that obesity is a risk factor for cardiovascular events, some studies have demonstrated better outcomes following cardiovascular events in obese patients, a phenomenon called the “obesity paradox” [14]. This finding was replicated in our study investigating the relationship between MetS and short-term morbidity and mortality outcomes among adult patients with STEMI treated with stenting. We found that STEMI patients with MetS had a significantly lower median cost of care and lower in-hospital mortality despite having higher rates of cardiovascular disease (CVD) risk factors, including diabetes, hypertension, hyperlipidemia, CKD, and obstructive sleep apnea. Curtis et al. found that individual components of the metabolic syndrome rather than the metabolic syndrome are important independent predictors of long-term medical costs, especially among elderly individuals [22].

The reduced mortality risk among MetS patients demonstrated in our study may be attributable to body anthropometry. In particular, increased adiposity reported in MetS patients may decrease the odds of death in STEMI patients with MetS following stenting through a series of mechanisms. First, obese individuals typically have greater accompanying lean mass, which may improve overall cardiorespiratory fitness, leading to better survival outcomes after heart failure and CAD [14]. It may also provide additional metabolic reserve in these individuals, as greater fat mass seems to be protective against the chronic catabolic state and cachexia characteristic in congestive heart failure (CHF), and other high-stress states, favoring better clinical outcomes, especially in the absence of systemic inflammation [14,23]. For instance, in a study by Anker et al., the cachectic state was shown to be an independent risk factor for mortality in patients with CHF, with about 50% of cachexic heart failure patients dying within 18 months of follow-up [24]. Similarly, overweight and obese individuals with heart failure and CAD were shown in other studies to have lower rates of hospitalization, all-cause and cardiovascular mortality rates than patients with lower body mass index (BMI) [2,25-27]. Furthermore, a meta-analysis of elderly subjects reported that overweight and obese individuals paradoxically had a lower risk of all-cause and cardiovascular mortality when compared to those with lower BMI, even though obesity is an independent CVD risk factor [28].

The obesity paradox has also been specifically reported in STEMI patients. Previous analyses of the National Cardiovascular Data Registry (NCDR) ACTION Registry-Get With The Guidelines (GWTG) database, which like our dataset predominantly consists of older adults, males, Caucasians, and >80% of those who had undergone a primary PCI, showed a U-shaped association between BMI and long-term mortality such that mildly obese patients with STEMI had the lowest mortality rates compared to normal weight and extremely obese patients, with the highest in-hospital mortality seen in class III obesity patients [29,30]. A similar U-shaped relationship was observed between BMI and MACE [29]. These favorable effects of mild obesity on mortality outcomes have also been reported in NSTEMI patients [31].

Consistent with the existing scientific literature, our study showed improved survival among patients with hypertension. This finding may also be explained by the “obesity paradox.” The International Verapamil Sustained Release (SR)-Trandolapril Study (INVEST) randomized trial demonstrated a lower risk of death, non-fatal MI, or non-fatal stroke in overweight and class I to III obese patients with hypertension compared to normal-weight participants [32]. Similarly, in the Antihypertensive and Lipid-Lowering Treatment to Prevent Heart Attack Trial (ALLHAT), obese BMI was associated with lower mortality rates in hypertensive patients [33].

While the obesity paradox has been observed to be consistent across racial-ethnic subgroups [34-36], there is emerging evidence for racial/ethnic and gender effect modification in the obesity paradox. For instance, greater protective effects of higher BMI on mortality outcomes have been reported among men undergoing cardiac surgery than women [37,38] and among African American and Hispanic populations [39]. This is particularly relevant given that over 65% of our study population were males, and 16% were identified as African American or Hispanic. Therefore, future prospective studies are needed to investigate the contribution and clinical relevance of race/ethnicity and gender on the prognosis of individuals with higher BMI with MetS.

It is also plausible that pre-and post-procedural severity or control of constituent risk factors of MetS may improve the prognosis of patients undergoing stenting procedures. Earlier studies have shown that baseline fasting blood glucose is an independent predictor of post-PCI restenosis irrespective of the glucose-lowering pharmacotherapy [40]. Similarly, a lower incidence of MACE, stroke and post-procedural complications has been observed in patients with optimal postoperative glycemic control [41,42]. In addition, high baseline triglyceride (TG)-glucose index, a surrogate marker of insulin resistance, has been associated with MACE, in-stent restenosis, and all-cause mortality [43,44], while elevated post-PCI non-high-density lipoprotein (HDL) cholesterol, a key component of dyslipidemia, has been associated with an increased risk of revascularization [45]. Furthermore, post-PCI hypertriglyceridemia [46] and TG/HDL-C ratio [47] or high systolic and diastolic blood pressure at the time of PCI have all been positively and independently associated with the risk of in-stent restenosis [48].

Limitations

Our study has the following identified limitations: as discussed above, pre-existing therapies for managing constituent risk factors of MetS may influence post-PCI survival outcomes, and because the NIS database does not include medication history, we were unable to include this in our analysis. Moreover, the impact of MetS on post-PCI outcomes is expected to emerge in the long-term, and our analysis is limited to in-hospital outcomes. Although prior long-term studies suggest favorable mortality outcomes in mildly obese STEMI patients, the long-term prognosis of STEMI patients with MetS remains unclear. Also, our study did not assess the impact of the type of stent used or the utilization of assistive imaging technologies such as intravascular ultrasound or optical coherence tomography. Finally, there is a potential risk of selection bias due to the non-random allocation of interventions, the risk of coding errors, including underutilization of MetS ICD codes, and missing data that is inherent to any large database study. However, the National Inpatient Sample auditing process is well-established, minimizing data inaccuracy issues. While unmeasured confounders may exist, they are expected to be evenly distributed among all groups.

## Conclusions

In conclusion, patients with MetS who underwent coronary stenting following STEMI have a significantly lower in-hospital mortality. The clinical implications of our findings are increasingly relevant given the rising trend of metabolic syndrome and the utilization of percutaneous coronary interventions in the US. We noted a higher prevalence of associated comorbidities, including hypertension, diabetes, and hyperlipidemia, but a lower prevalence of heart failure and COPD in patients with MetS compared to the non-MetS groups. The components of MetS, gender, and race/ethnicity that may have contributed to the observed reduction in mortality remain unclear. Future randomized control trials are needed to evaluate the nuanced associations.
